# Perinatal Resilience for the First 1,000 Days of Life. Concept Analysis and Delphi Survey

**DOI:** 10.3389/fpsyg.2020.563432

**Published:** 2020-11-03

**Authors:** Sarah Van Haeken, Marijke A. K. A. Braeken, Tinne Nuyts, Erik Franck, Olaf Timmermans, Annick Bogaerts

**Affiliations:** ^1^Research & Expertise, Resilient People, UC Leuven-Limburg, Diepenbeek, Belgium; ^2^Faculty of Medicine, Department of Development and Regeneration, Women and Child, KU Leuven, Leuven, Belgium; ^3^Faculty of Rehabilitation Sciences, REVAL-Rehabilitation Research Center, Hasselt University, Diepenbeek, Belgium; ^4^Faculty of Medicine and Health Sciences, Centre for Research and Innovation in Care (CRIC), University of Antwerp, Antwerp, Belgium; ^5^Professorship Healthy Region, HZ University of Applied Sciences, Vlissingen, Netherlands

**Keywords:** resilience (psychological), perinatal mental health, perinatal care, parenthood, childbirth

## Abstract

**Background:**

The ability to cope with challenges and stress in life is generally understood as resilience. Pregnancy and parenthood are challenging times. The concept of resilience is receiving increasing interest from researchers, clinicians, and policy staff because of its potential impact on health, well-being, and quality of life. Nevertheless, the concept is less studied during the perinatal period.

**Objectives:**

The aim of this study is to understand the concept of perinatal resilience, including the underlying processes and more specifically for the first 1,000 days of life.

**Methods:**

A concept analysis according to the Walker and Avant (2011) framework was used, to investigate the basic elements of the concept. Concurrently, a two-round Delphi survey involving researchers, clinicians, epidemiologists, mothers, and fathers (*N* = 21), was conducted to prioritize the terms associated with perinatal resilience. Data collection took place between January and April 2019.

**Results:**

Through concept analysis and Delphi survey, five defining attributes for perinatal resilience were identified: social support, self-efficacy, self-esteem, sense of mastery and personality. The additional terms, rated important by the Delphi survey, were linked to the consequences of being resilient during the perinatal period for the individual and his/her family. Specifically, highlighted were the experiences of families in personal growth and achieving family balance, adaptation, or acceptance.

**Conclusion:**

Based on the results of the concept analysis and Delphi survey, we describe perinatal resilience for the first 1,000 days as a circular process towards a greater well-being in the form of personal growth, family balance, adaptation or acceptance, when faced with stressors, challenges or adversity during the perinatal period. The presence of resiliency attributes such as social support, sense of mastery, self-efficacy, and self-esteem enhance the capacity to be resilient and probably prevent mental health problems.

## Introduction

In the past few years, the concept of resilience has been receiving increasing interest from researchers, clinicians, and policy staff. This interest is largely due to the potential impact resilience can have on health, well-being and the quality of life (Powley, 2009; Windle, 2011).

The concept of resilience was originally applied within physical sciences (Barasa et al., 2018). In the 70’s, early psychiatric literature developed the concept of “psychological resilience” based on the examination of children who were exposed to adverse life situations (e.g., poverty) (Earvolino-Ramirez, 2007). Meanwhile, a broad range of disciplines explored the concept of “resilience” in different contexts such as family abuse, chronic illness and eating disorders (Harrop et al., 2006; Earvolino-Ramirez, 2007). Given the increasing amount of research in several disciplines, different definitions, and conceptualizations of resilience have been published across clinical and scientific literature (Gallopin, 2006; Fletcher and Sarkar, 2013). This haziness leads professionals to create a diversity of interpretations about the meaning of the concept “resilience”. Resilience is generally understood as the ability to cope with the challenges, stresses, and adversities in life (Waugh and Koster, 2015).

Pregnancy and new parenthood are challenging times with many emotional, physical and social changes to the mother, her partner and their surroundings. Some of the pregnant women and mothers find the experience of a pregnancy and the prospect of a new family member with the changes that occur exciting and joyous. Others experience a wide range of positive and negative emotions, which can result in biopsychosocial distress (Currid, 2004; Henderson and Redshaw, 2013). Similar, fathers (to be) can also experience stress due to negative feelings about the pregnancy, role restrictions related to parenthood, fear of childbirth, social isolation and self-efficacy about infant care (Baldwin et al., 2018; Young et al., 2018). The first 1,000 days of life refer to the period from conception to the age of the child up to 2 years. During this period, the fetus and later the infant are most adaptable, but also most vulnerable. Biological and developmental functioning of the offspring are affected by genetics (conception) and by environmental influences such as maternal stress, nutritional state, and social network (O’Reilly and Reynolds, 2013). This critical period is seen as an important life phase in the prevention and development of chronic physical and mental disorders in adulthood and beyond, such as depression, (childhood) obesity, and cardiovascular diseases (Catalano and Shankar, 2017; Koletzko et al., 2019).

An increasing body of research focused on the identification of resilience-promoting characteristics and mechanisms, which can be beneficial for mental health outcomes (Schiele and Domschke, 2018). However, only a few studies assess resilience during the perinatal period, with most of them focusing on unusual trajectories with the presence of adversity or trauma such as intimate partner violence or teen pregnancy (Wilson-Mitchell et al., 2014; SmithBattle and Freed, 2016; Dekel et al., 2017; Barnett et al., 2018; Xuemei et al., 2019). Yet a definition of perinatal resilience is absent. The focus of this research is however broader by considering every pregnancy as a major life-event accompanied by changes, challenges and stressors for the (expectant) mothers and the wider family. Becoming a mother or father is a developmental event that implies a new transition. This period is characterized by uncertainty, increased responsibility, sleep deprivation, a new role as parent and a re-establishment of the couple’s relationship (Serçekuş and Başkale, 2016).

Consequently, our aim is to explore the concept of perinatal resilience, including the underlying processes, and more specifically for the first 1,000 days of life.

## Methods

A concept analysis and Delphi survey are used because these methods are applicable and relevant to vague concepts that are prevalent in practice and have been used across disciplines (De Bleser et al., 2006; Earvolino-Ramirez, 2007).

### Concept Analysis

Concept analyses are used to examine the basic elements of a concept to investigate its structure and function (Walker and Avant, 2011). To guide this concept analysis, the Walker and Avant (2011) framework was selected because of its structured method and international use for healthcare concept analyses (Ridner, 2004). This framework includes the following eight steps: (1) concept selection; (2) determination of the aim of analysis; (3) identification of the uses of the concept; (4) determination of the defining attributes; (5) construction of a model case; (6) construction of additional cases; (7) identification of antecedents and consequences; and (8) definition of empirical referents.

**FIGURE 1:**
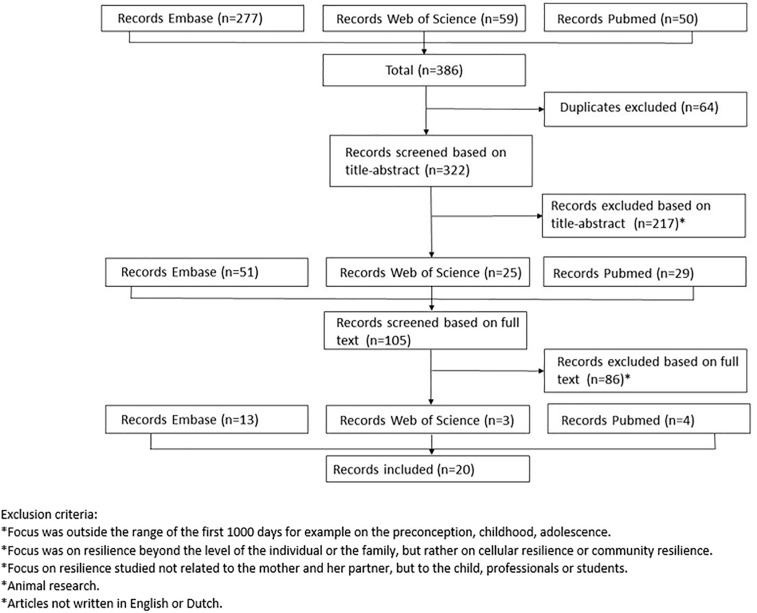
Flow chart of article selection.

In order to determine the attributes, antecedents and consequences of perinatal resilience, a search in PubMed, Embase and Web of Science was performed. The team consulted with a research librarian of the KU Leuven, who suggested a broad approach for designing the search given the exploratory nature of the study. MeSH (Medical Subject Headings) terms, Emtree terms and truncation were used in order to capture relevant studies. The search strings are attached in appendix A. The initial search strategy yielded a total of 386 results. We excluded studies outside the range of the first 1,000 days of life (e.g., preconception), studies focusing on resilience beyond the level of the individual (e.g., community resilience), studies focusing on resilience in children, adolescents, professionals or students, animal studies and studies not written in English or Dutch. After removing duplicates and applying the exclusion criteria, 217 studies were excluded. We then screened the full text of the remaining 105 studies and found a further 86 which met the exclusion criteria. The procedure used for article selection is as described in Figure 1. The selection process was carried out by two independent assessors (SVH and TN). No quality assessment of the individual papers took place. This was because we focused on the way resilience was described, defined and used instead of the “findings” from the studies. Ultimately, 20 studies were included in the analysis.

Thereafter each article was individually screened for the presence of perinatal resiliency attributes. Walker and Avant (2011) define attributes as being present characteristics frequently associated with the concept. Attributes related to resilience were listed within an article and this for each article separately. Afterward, the attributes were put together in one file. In the next step, synonyms and linked attributes were regrouped by theme. In total 12 groups were formed. Attributes were counted per theme resulting in a sum score per theme. Groups with a sum score higher than five were selected for the further development of the description of perinatal resilience. The selection of the attributes was an inductive, cyclical process with constant dialogue between the members of the research team (SVH, MAKA, and AB).

### Delhpi Survey

A two-round Delphi survey (January–April 2019) was conducted to supplement the concept analysis. A Delphi panel was convened to contribute input to the concept of perinatal resilience and prioritize the terms associated with perinatal resilience. We sought to recruit a varied panel of experts who were familiar with the field of pregnancy, childbirth and parenthood, had published about resilience or had experience within the field. In line with the recommendation of Linstone and Turrof, we recruited 21 experts from Belgium (*N* = 20) and the Netherlands (*N* = 1). The panel consisted of researchers (*N* = 10), clinicians (*N* = 13), and epidemiologists (*N* = 2). Most of the panel are also experienced as mothers or fathers.

The Delphi survey was designed to explore the range of opinions within the theme of resilience with the aim to achieve consensus about the topic. Based on the reviewed literature for our concept analysis, a survey was created that listed resilience relating terms (*N* = 89).

In the first round, this survey was sent to all participants of the panel with the request to score each of the identified terms for their relevance in perinatal resilience. A nine-point Likert scale was used to evaluate the importance of each term. A score of 9 is considered to be critical with score of 1 deemed of limited importance. Participants also had the opportunity to add terms to the original list. Experts were then asked to return their responses to the second author. In the second round, the survey was returned to each individual expert containing group scores and their individual scores for each item. We provided the median and interquartile range for each term which reflects their importance and unanimities as assessed in the first Delphi round. Participants were invited to review their individual ratings against the scores of the group. They then resubmitted their responses changed or unchanged to the second author. Based on the first round, six terms were added to the original list. Non-responders (*N* = 1) from the first round were not invited to the subsequent round. Group median scores were calculated for each item and then ranked from highest to lowest. Terms with a median score above the predefined threshold of eight or more were assumed to be critically relevant terms associated with resilience during the perinatal period. Only those were chosen for further evaluation of the description of the concept of perinatal resilience. The ranking represented the group’s consensus. We maintained full anonymity between experts, and the complete results were only known to the pollster (MAKA).

## Results

### Results Concept Analysis

The first step of the Walker and Avant (2011) method is to select the concept of interest. In this research the focus is specifically on perinatal resilience for the first 1,000 days of life. The second step is to define the aim of the analysis, which is to clarify the meaning of the concept perinatal resilience within the context of the first 1,000 days of life and its underlying processes, in order to better understand and apply the mechanism by which perinatal resilience is able to promote health and well-being during the perinatal period.

#### Identifying Uses of the Concept

The third step is to identify the various uses of the concept by consulting different resources such as dictionaries, thesauruses and research databases (Walker and Avant, 2011).

The Oxford English Dictionary (2018) defines “perinatal” as “*an adjective relating to the time, usually a number of weeks, immediately before and after birth (Perinatal, 2018)*.” However, there is some dispute about the exact time frame the perinatal period covers.

The term resilience derives from the Latin “resilentia”/“resilire,” which means “*to jump*” or “*to bounce back*” (Windle, 2011). The Oxford English Dictionary (2018) defines “resilience” as (1) “*the capacity to recover quickly from difficulties; toughness*” or (2) “*the ability of a substance or object to spring back into shape; elasticity (Resilience, 2018b).*” The Merriam-Webster Dictionary (2002) defines resilience as “*an ability to recover from or adjust easily to change or misfortun*e” or “*the capability of a strained body to recover its size and shape after deformation caused especially by compressive stress (Resilience, 2018a).*”

The concept “resilience” has its origins in the field of physics and mathematics (Norris et al., 2008; Reid and Botterill, 2013). Initially resilience referred to the *“ability of a strained body, by virtue of high yield strength and low elastic modulus, to recover its size and form following deformation”* (Norris et al., 2008; Reid and Botterill, 2013). Within these disciplines, it is implicitly assumed that a system returns to an equilibrium after the presence of some kind of disturbance. From an ecological perspective, resilience is the ability of an ecosystem to absorb shocks while maintaining function (Masten and Powell, 2003).

The origin of the concept of psychological resilience stems from the early psychiatric literature that examined children who appeared to be invulnerable to adverse life situations (Hunter, 2001; Tusaie-Mumford, 2001; Henry, 2002; Pilowsky et al., 2004). A study of Werner and Smith (1982) with 698 individuals, showed that of the children that grew up in poverty or other adverse conditions (e.g., parental divorce, alcoholism, or mental illness), approximately two-thirds of these children developed serious problems as adults. One-third developed into competent, caring adults (Werner, 1996). These results encouraged research into psychological resilience (Earvolino-Ramirez, 2007). The meaning of resilience shifted to a process of growth and adaptation rather than a state of bouncing back after experiencing adversity or challenges (Richardson, 2002).

Recently, research has expanded to other disciplines such as midwifery, nursing and medicine (Caldeira and Timmins, 2016; Goemaes et al., 2016). In these studies, resilience is linked to several mental health problems such as posttraumatic stress disorder (PTSD) and specific populations such as breast cancer survivors, elderly and cardiac stent placement patients (Seng et al., 2011; Sexton et al., 2015; Zhang et al., 2017; Calvete et al., 2018; Dikmen-Yildiz et al., 2018). Others have focused more on the community level rather than perceiving resilience as an individual attribute. Social or community resilience was defined by Adger (2000) as “*an important component of the circumstances under which individuals and social groups adapt to environmental change*” (pp. 347).

Although the context of the concept may change, the concept of resilience across all of these fields is closely related with the capability to return to a stable state after a disruption (Bhamra et al., 2011). In some disciplines, resilience implies a capacity to return to the equilibrium while in others, it refers to the individual capacity to adapt or even to the potential for growth. Some state that resilience can be understood as a fixed personality trait whilst some define resilience as a dynamic process (Southwick and Charney, 2012; Johnston et al., 2015; Niitsu et al., 2017). Given the popularity of the concept within various disciplines, a wide range of definitions is available (Fletcher and Sarkar, 2013).

To narrow the search for this concept analysis, the focus is on personal resilience and more specifically on perinatal resilience. Research about resilience within the perinatal period has mainly focused on the presence of adversity or trauma such as intimate partner violence, teen-pregnancy and postpartum depression (Wilson-Mitchell et al., 2014; SmithBattle and Freed, 2016; Dekel et al., 2017; Barnett et al., 2018). Suspected components of resilience have been associated with improved perinatal outcomes, such as increased birth weight, decreased rates of postpartum depression, emotional stress and sleeping problems (Roos et al., 2013; Waugh and Koster, 2015; Hain et al., 2016; Johnson et al., 2018). However, pregnancy and parenthood can’t be defined as an adversity but it can certainly be described as a major life-event. Also parents without significant additional risk factors experience distress during pregnancy, childbirth and parenthood and perceive this as a challenging time (Young et al., 2020). The presence of resilience characteristics can help (future) parents to positively adapt to this new situation and role (Fletcher and Sarkar, 2013).

#### Defining Attributes

**FIGURE 2:**
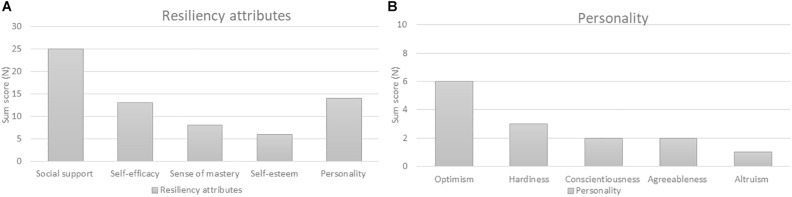
**(A)** Sum scores of resiliency attributes based on a concept analysis. **(B)** Sum scores of personality traits linked to perinatal resilience based on a concept analysis.

Walker and Avant (2011) described defining attributes as being present characteristics frequently associated with the concept. These attributes allow a broad insight into the concept. The following attributes were compiled after doing an extensive screening about perinatal resilience in the included articles (*N* = 20). In consultation with the research team these five groups were selected as perinatal resiliency attributes based on their sum scores (see Figure 2).

##### Social Support

Social support is a very common term used throughout perinatal resilience literature, with a sum score of 25 (Schachman et al., 2004; Callahan and Borja, 2008; Gagnon and Stewart, 2014; Noyman-Veksler et al., 2015; Ramey et al., 2015; Hain et al., 2016; Maxson et al., 2016). Social support can be divided in different sources of support such as paternal support, spousal or partner support, support from family and friends, professional and community support. The social support system for (expectant) mothers and fathers has been identified as an important influencer on resilient outcomes. Maxson et al. (2016) examined how psychosocial health, prenatal health and pregnancy outcomes are associated within a sample of 1,499 pregnant women. They identified three psychosocial health profiles which were labeled as “resilient” (*N* = 509), “vulnerable” (*N* = 278) and “moderate” (*N* = 526). Women in the “resilient” category are characterized by low levels of depression and perceived stress, high interpersonal (*M* = 43.82) and paternal support (*M* = 2.78) in comparison with the “vulnerable” group (interpersonal support *M* = 28.51; paternal support *M* = 2.37). Recent studies report that the simple presence or absence of a support network is predictive of emotional reactions and may buffer the negative effects of stress and depression (Callahan and Borja, 2008). Several studies also show that social support is an important factor in building resilience and can be considered a protective factor during this stage of life (Nichols and Roux, 2004; Alderdice et al., 2013; Mautner et al., 2013).

##### Self-Efficacy

Various studies cite “self-efficacy” as a contributor to prenatal psychosocial health (sum score of 13) (Alderdice et al., 2013; Maxson et al., 2016). Self-efficacy is described as the belief in one’s capability to achieve a goal or overcome an event (Bandura, 1982; Earvolino-Ramirez, 2007). Maxson et al. (2016) assert that women with more vulnerable psychosocial health profiles such as low self-efficacy (*M* = 2.80) and low social support, are more likely to have risky health correlates (e.g., substance use) before and during pregnancy. Women with a resilient health profile have higher levels of self-efficacy (*M* = 3.64, *p* < 0.05). This is in line with research of Li et al. (2016), they concluded that women with high resilience possess positive characteristics such as self-efficacy which can help them to adapt in the face of acute or chronic stress. Self-efficacy is also a predictive factor for increased birth satisfaction (*r* = 0.19, *p* < 0.01) (Berentson-Shaw et al., 2009).

##### Sense of Mastery

A third perinatal resilience factor is “sense of mastery,” which receives a sum score of eight (Britton, 2005; Handelzalts et al., 2016). Sense of mastery is defined as finding strength and meaningfulness following a stressful experience, adversity or trauma (Elmir, 2014). Sense of mastery is described as a robust stress-resistance resource (Britton, 2005; Maxson et al., 2016). Research demonstrate that mastery is significant in predicting physical and mental health in stressful situations. Britton (2005) observed a significant inverse correlation between anxiety scores and a sense of mastery (*r* = −0.44, *p* < 0.001). Women with adaptive resources, such as a positive sense of self and mastery, have healthier pregnancy and birth outcomes (Maxson et al., 2016). Additional research also showed a positive relation between a sense of mastery and the postpartum psychiatric functioning (*p* < 0.001) (Eshbaugh, 2010; Sexton et al., 2015).

##### Self-Esteem

Self-esteem compared to the attributes above is linked to perinatal resilience with a sum score of six. Self-esteem can be paraphrased as a positive or negative perspective toward oneself (Hajek and König, 2019). Self-esteem is marked as a buffer against stress and adversity (Harrop et al., 2006; Johnson et al., 2018). Pregnant women with high levels of self-esteem, mastery, and optimism appear to have low levels of perceived stress (Maxson et al., 2016). Research from Mautner et al. (2013) indicates that a depressive condition after childbirth is characterized by reduced self-esteem, anxiety and sleep difficulties. By focusing on the self-esteem of pregnant women and young mothers, we can strengthen resilience which in turn is a potential protective factor for ante- and postpartum depression (Harrop et al., 2006; Gagnon and Stewart, 2014).

##### Personality

A final influencing factor of the perinatal resilience process is personality with a sum score of 14 (Mautner et al., 2013; van der Zwan et al., 2017; Johnson et al., 2018). A common personality trait is optimism. Optimism can be defined as the generalized expectancy that good outcomes will occur when confronting major problems (Handelzalts et al., 2016). Optimism is considered as a protective factor against prenatal anxiety (Alderdice et al., 2013; van der Zwan et al., 2017). Other resilience-related personality variables are hardiness, conscientiousness, agreeableness and altruism. Those variables appear to promote resilience (Callahan and Borja, 2008; Gagnon and Stewart, 2014).

#### Model Case and Additional Cases

In the next part a model case and additional cases are described. A model case is an example of the use of the concept that demonstrates all the defining attributes of the concept (Walker and Avant, 2011). The additional cases consist of a borderline case and a contrary case. The borderline case contains most of the defining attributes of the concept being examined but not all of them and the contrary case contains the defining attributes in the opposite way (Walker and Avant, 2005). The cases are hypothetical, based on the author’s experience in clinical practice.

##### Model Case

Marie, an optimistic primipara woman of 30, is in active labor with cervix dilation of 7 cm. She is supported by her husband and midwife but gets a panic attack following on the painful contractions. In panic she screams that the contractions are very painful and she can’t handle them. Her midwife and partner frame the feelings she experienced as a good sign with the labor proceeding positively. Her husband replies to her that she is a strong woman who is doing well and that she is capable to cope with the pain. This helps Marie to make sense of the pain and the emotions she is feeling. To support her, her husband says “Keep going, you are doing well!” Marie also remembered the sessions she attended during her pregnancy and used the techniques that she learned there about breathing and relaxation.

Present resiliency attributes: social support (e.g., husband), sense of mastery (e.g., make sense), self-efficacy (e.g., techniques about breathing and relaxation), self-esteem (e.g., strong woman) and personality (e.g., optimistic).

##### Borderline Case

Ellen is a woman of 27 years and married for over 5 years to her partner. Together they look forward to the arrival of their second child. Ellen is currently 30 weeks pregnant. She is uncertain and worried because her first child was born 7 weeks premature. Also a close friend of Ellen had an emergency cesarean section. The realization that something can go wrong, causes stress and anxiety to Ellen and her partner. They are both concerned about the health status of their baby and Ellen isn’t sure she can deal with unforeseen circumstances. Ellen attends a psychoeducation session about stress to help her recognizing and coping with her stress signals. Fortunately, Ellen can count on her family and friends and discuss these worries with them.

Present resiliency attributes: social support (e.g., family), self-efficacy (e.g., psychoeducation about stress).

##### Contrary Case

Cathy is a young woman of 18 years. She became pregnant which was unplanned with her former boyfriend. He left her when she was 6 months pregnant. Now almost 1 year later, she is living with her parents and her 9 month old son. Until now her parents have taken care of her son and Cathy has finished her studies. Cathy starts working at a department store and she is searching for a place of her own. Cathy is anxious and insecure about the future. Is she ready to raise a child on her own with no partner on her side? Is she capable of being a good mother? Will she earn enough money to live on her own and provide a safe home for her son? A lot of questions to which Cathy has no answer and she feels she has already failed.

Except the support of her parents, no resiliency attributes are present.

#### Antecedents and Consequences

According to Walker and Avant (2011), defining the antecedents and consequences is an important step to understand the concept within the social context. Antecedents are the triggers such as an event or incident that must occur prior to the occurrence of the concept. Consequences are those events that occur as a result of the appearance of the concept (Walker and Avant, 2011).

##### Antecedents

In case of perinatal resilience, we search for triggers that are categorized as “resiliency challenges.” First, pregnancy, childbirth and future parenthood can be considered as life events with the presence of changes, challenges, and stressors (Currid, 2004; Henderson and Redshaw, 2013). Examples of concurrent stressors are lack of sleep, adjustment to increased responsibilities, new role as a parent, re-establishment of the partner relationship and return to the workplace (Nichols and Roux, 2004; Mizukoshi et al., 2016). Second, during the perinatal period situations may occur that go beyond the daily hassles and are labeled as an adversity or traumatic event. Examples include complications during pregnancy or traumatic childbirth which may activate the presence of resiliency attributes (Callahan and Borja, 2008; Dikmen-Yildiz et al., 2018; Wilson and Cook, 2018). Third, it is important that the individual perceives the situation as stressful or challenging to activate the resiliency attributes (Franklin et al., 2012; Dekel et al., 2017).

##### Consequences

Pregnancy, childbirth, and parenthood are emotionally charged events which can elicit acute or chronic distress. The perinatal period therefore has a number of pitfalls that have an influence on mental well-being. One of the most frequent mental health problems during the perinatal period is ante- and postpartum depression (PPD). Approximately 7% of women suffer from major depression in the first 3 months after delivery and the prevalence increases to 19.2% with minor depression included (Gavin et al., 2005; Molenaar et al., 2018). The prevalence rate of depression by fathers is 10.4% between the first trimester of their partner’s pregnancy and 1 year postpartum (Paulson and Bazemore, 2010). Also anxiety disorders occur frequently, with a prevalence of approximately 20% for women and 4.1–18% for men during pregnancy and postpartum period (Leach et al., 2016; Fawcett et al., 2019). A smaller group of women suffer from more severe conditions such as psychosis (1–2 per 1,000) or post-traumatic stress disorder (PTSD) (3%) (Grekin and O’Hara, 2014; Howard et al., 2014).

Multiple studies show the preventive nature of the attributes of perinatal resilience regarding psychological outcomes. Future parents with access to resiliency attributes at the time of stress or adversity during the perinatal period have more chances to deal with these stressors in a positive way. In this case, the perinatal period can be a catalyst toward personal growth, family balance, well-being, adaptation or adjustment with a higher experienced quality of life (Nichols and Roux, 2004; Callahan and Borja, 2008; Mautner et al., 2013; Elmir, 2014; Takegata et al., 2014; Irwin et al., 2016; Mizukoshi et al., 2016; van der Zwan et al., 2017).

#### Empirical Referents

In a final step of the concept analysis, researchers determine the empirical referents for the defining attributes (Walker and Avant, 2011). According to Walker and Avant “*empirical referents are defined as the categories or groups of actual phenomena that, by their existence, demonstrate the occurrence of the concept itself*”. This last part of the concept analysis focuses on how “perinatal resilience” could be measured. Currently there are some empirical instruments available to recognize or measure the occurrence of resilience. A review of Windle et al. (2011) of 19 resilience scales noted that the Connor-Davidson Resilience Scale, the Resilience Scale for Adults and the Brief Resilience Scale possess the best psychometric ratings. However, perinatal resilience has specific features that differ from resilience in general and to our knowledge no instruments are available to measure resilience during the perinatal period. To assess perinatal resilience, it might be interesting to measure the defining attributes as separate and additional constructs.

### Results Delphi Survey

In the first round seven terms had a median score of ≥8 and were considered critically relevant to perinatal resilience: social support, self-efficacy, adaptability, self-confidence, coping style, having a supportive person and to put into perspective. The second round resulted into the same set of terms with the addition of a positive self-image as critically relevant to perinatal resilience. The scoring between the first and second round was congruent overall (see Figure 4).

## Discussion

There is an increasing body of research into resilience as an essential component of health and well-being (Herrman et al., 2011). Perinatal mental health problems such as anxiety or depression (14–25%) are common with prevalence rates coming close to the rates of prenatal medical complications such as gestational diabetes and hypertension (Milgrom et al., 2008; Kingston et al., 2014, 2015). Resiliency attributes have been associated with improved perinatal outcomes and can be beneficial in the prevention of perinatal mental health problems (Johnson et al., 2018). The aim of this paper was to review the concept of perinatal resilience for the first 1,000 days of life.

Through a concept analysis we identified five defining attributes of perinatal resilience: social support, self-efficacy, sense of mastery, self-esteem, and personality. Social support and self-efficacy were also rated majorly important in the Delphi survey. Additionally, the Delphi survey indicated self-confidence, having a positive self-image and supportive person as being critically relevant to perinatal resilience. They are strongly related to self-efficacy, self-esteem, and social support. To put into perspective can be seen as part of sense of mastery as both refer to the allocation of a meaning. Adaptability and coping style were other outcomes of the Delphi survey, which can then be considered as consequences of resilience.

Earlier resilience research within this context focused mainly on family resilience and mostly within the context of unusual circumstances such as illness, intimate partner violence or teen pregnancy (Young et al., 2018). Our work contributed to this gap in the literature by focusing on a general population rather than (expectant) parents in “at risk” groups. The absence of significant risk factors in (future) parents, doesn’t mean they don’t experience distress during pregnancy, childbirth and parenthood and perceive this as a challenging time (Young et al., 2020). Current study made clear that challenges, stress, and adversity are antecedents of resilience. Being resilient during the perinatal period has many positive consequences such as personal growth, family balance, well-being, adaptation, or adjustment (Nichols and Roux, 2004; Callahan and Borja, 2008; Mautner et al., 2013; Elmir, 2014; Takegata et al., 2014; Irwin et al., 2016; Mizukoshi et al., 2016; van der Zwan et al., 2017). Perinatal resilience can play a major role in buffering against antepartum and postpartum depression and anxiety (Takegata et al., 2014; Sexton et al., 2015; Hain et al., 2016; Irwin et al., 2016) and has a positive influence on the quality of life (Mautner et al., 2013; van der Zwan et al., 2017). When perinatal resiliency attributes are absent, some negative consequences may occur such as perinatal anxiety or postpartum depression.

A difficulty that arises within this research is the perinatal timeframe. The perinatal period is, based on historical grounds, defined as a number of weeks immediately before and after birth. However, according to the research team this definition is insufficient within a health-promoting and preventive approach. We therefore underscore the first 1,000 days of life because of the relevance of this established concept within prevention and health promotion research. The period of conception to 2 years after birth is key in the development of children throughout life and an important life phase in the prevention and development of chronic physical and mental disorders in adulthood (Catalano and Shankar, 2017; Koletzko et al., 2019). Also resilience research wants to identify specific periods of acute developmental change to maximize the efficacy of later interventions (Luthar et al., 2000; Young et al., 2018). Previous research found that pregnancy and the transition to parenthood is a vulnerable time accompanied with challenges which require a resilient response (Gavidia-Payne et al., 2015; Young et al., 2018).

**FIGURE 3:**
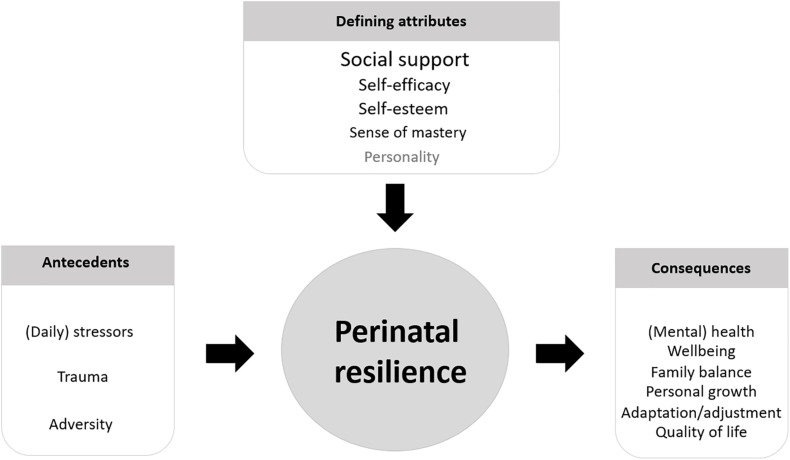
Perinatal resilience for the first 1,000 days of life.

Following our analysis of the results, we propose a description of perinatal resilience for the first 1,000 days of life as follows: *perinatal resilience is a circular process toward a greater well-being in the form of personal growth, family balance, adaptation, or acceptance, when faced with stressors, challenges, or adversity during the perinatal period. The presence of resiliency attributes such as social support, sense of mastery, self-efficacy, and self-esteem enhance the capacity to be resilient and prevent mental health problems (see Figure 3).*

**FIGURE 4:**
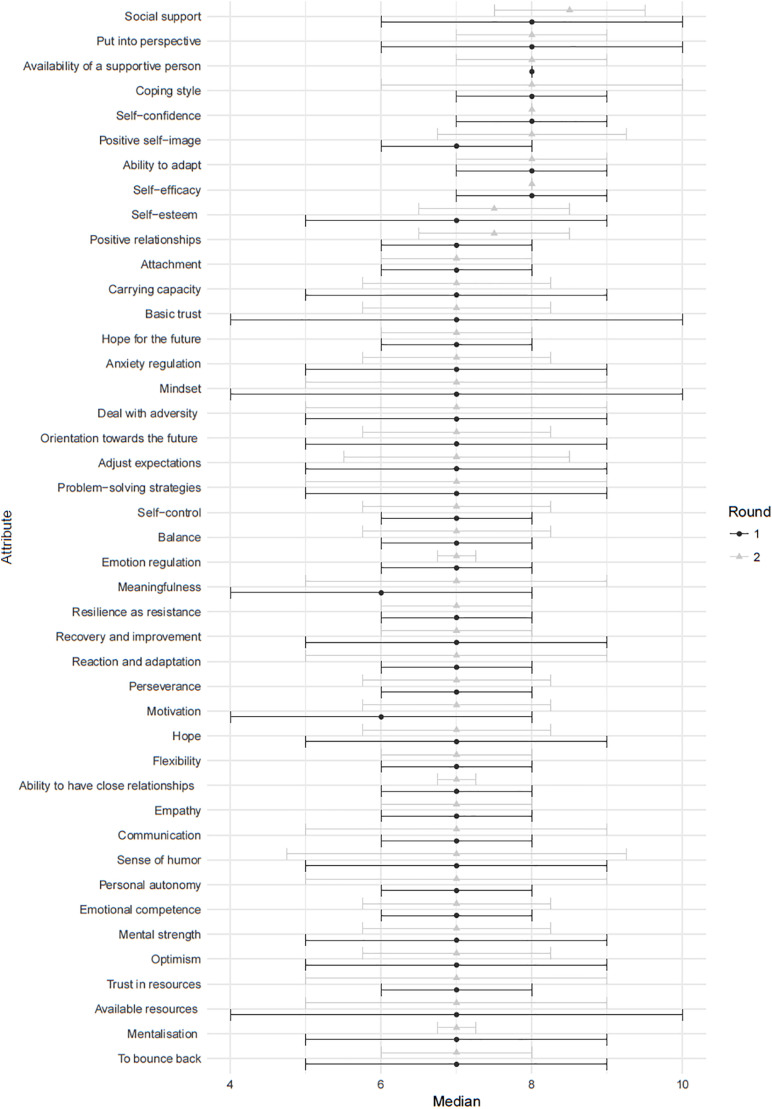
The results of round 1 and 2 of the Delphi survey.

Some of our results confirmed findings from other studies on resilience. The synthesis of Caldeira and Timmins (2016) about concept analyses of resilience, indicates that the antecedents of resilience are an adverse situation, changes, resources, and the awareness of the circumstances. We also found similarities in case of defining attributes. Social support is often mentioned as an attribute (Windle, 2011; Garcia-Dia et al., 2013; Caldeira and Timmins, 2016). Self-efficacy and self-esteem were additional attributes mentioned in other general resilience research (Windle, 2011; Garcia-Dia et al., 2013).

Our findings are in line with the current trend that resilience is a dynamic, modifiable process (Luthar et al., 2000; Reich et al., 2010; Southwick and Charney, 2012; Johnston et al., 2015). We demonstrate that perinatal resilience is the product of a dynamic process between the individual (e.g., personality) and his or her environment (e.g., social support). The extent to which someone can be resilient will fluctuate over time and is intensively influenced by the context (Garcia-Dia et al., 2013).

Our data also generated some complimentary knowledge above the general resilience research. A sense of mastery seems to be a specific attribute of perinatal resilience. This includes the ability to make sense of circumstances in the face of stressful events (Elmir, 2014), to put something in perspective and the possibility to discuss difficulties and reach a point of meaningfulness. There are some similarities with the construct “sense of coherence” of Antonovsky (1996) and more specifically to the key element “meaningfulness.” The concept of “sense of coherence” or SOC fits within the theories about health promotion and provided a theoretical framework for salutogenic research (Antonovsky, 1996; Harrop et al., 2006).

Similar to SOC, resilience is a key concept within positive psychology and salutogenesis. Both focus on factors that support human health and well-being, with the focus on strengths instead of weaknesses. A focus that forms an interesting starting point within health promoting and preventive research. However, until now resilience for the perinatal period and the first 1,000 days of life has remained vague with no clear indication how to enhance resilience during this period. Current study answers this need with a concrete description of the concept. This can lead to a practical translation of the resiliency attributes into a resilience enhancing intervention. This in turn can contribute to a broader sense of coherence. Both fit within a salutogenic framework. Nowadays, the paradigm shift from being disease-oriented to a salutogenetic framework, is going on within healthcare and health policy. Knowing that resilience is a dynamic process that can be influenced is an important finding for healthcare practices. The use of the concept “resilience” in future interventions can support professionals in taking a lead in the salutogenic approach of health and well-being and can be an innovative element in the current prevention policy. Enhancing resilience early in pregnancy, may reduce subsequent disorders in the mother, the child and the partner.

### Strengths and Limitations

#### Strengths

First, the structured eight step framework of Walker and Avant used and described in detail in this study increases the replicability of the study. The transparent inclusion and exclusion criteria with the use of a second independent assessor (TN), minimize the possibility of selection bias. Various sources of literature were incorporated and diverse uses of the concept were identified through exploring resilience within different disciplines. A concept analysis is part of theory development and makes the meaning of a concept explicit so it can be part of testable and practical theories. This paper utilized hypothetical examples but inspired by the experiences of the research team to illustrate examples of model, borderline and contrary cases of perinatal resilience.

Second, the Delphi survey improves the validity of our study as it complements the literature-based concept analysis with results that stem from an expert group opinion which is more valid than a decision made by a single person. The researchers also established trustworthiness by using an iterative approach and a detailed description of the Delphi collection and analysis process. Furthermore, although the Delphi survey and concept analysis were conducted by two different researchers, independently from each other, the results of both approaches are in line with each other and support the overall conclusion of our study. Three terms indicated by the Delphi survey match attributes identified by the concept analysis. Three other terms suggested by the Delphi survey were strongly connected to attributes advocated by the concept analysis.

Third, current studies recommend that the concept of resilience needs to be studied and coupled to a relevant domain outcome (Vanderbilt-Adriance and Shaw, 2008). Therefore we analyzed resilience within a specific group for a specific period of life. Despite the large volume of resilience research, the domains of pregnancy, childbirth and future parenthood remains underexposed. With this study we are the first to analyze resilience specifically focusing on the first 1,000 days of life. Our innovative findings are supplemental to existing research because it gives an overview and state of the art of the attributes specifically related to perinatal resilience for the first 1,000 days.

Fourth, this study adds important value in the current trends in health care policy, with attention to a more health-promoting and preventive approach. Resilience as a concept appears to be relevant in health context and prevention of mental health problems (Caldeira and Timmins, 2016) to face changes, challenges and stressors which parents may face during the perinatal period. Attention to psychological aspects within perinatal care is necessary because it not only affects the mother but also her infant and the rest of the family (Caldeira and Timmins, 2016).

#### Limitations

Some limitations of this study should be addressed. This study is not a systematic review or meta-analysis. Consequently, no quality assessment of included studies or statistical analyses were performed. Within our search strategy we only included articles that specifically mentioned the concept of resilience. A possible disadvantage is that we missed articles with resilience-relating concepts such as empowerment or articles mentioning resiliency attributes without using the concept of resilience as such. Another disadvantage, possibly linked to our search strategy is the used timeframe within the articles. Although we used the first 1,000 days of life as an inclusion criteria for article selection, the selected studies mainly focus on childbirth and the first weeks postpartum. A possible explanation for this is that research into resilience during pregnancy, childbirth and parenthood has not yet applied the 1,000 days of life framework. Because of this, we have to be careful regarding interpreting the results of the outcome section. Although, the most important attributes of the concept of perinatal resilience are covered in the methods used to gather results.

Another limitation is the absence of service users within this study. The concept analysis is based on studies retrieved by professional and academic databases. The participants of the Delphi study were healthcare providers, academics and epidemiologists. Despite the fact that almost all participants of the Delphi survey are a parent themselves, we can’t assume that they completed the survey from their parent role. It is more likely that they filled in the survey as an expert in working with parents during pregnancy, childbirth and postpartum. It is possible that our description of perinatal resilience isn’t accepted by the service users. A recent study of Young et al. (2020) performed a thematic analysis of parents’ perspectives of resilience experiences within the first year of parenting. In line with our findings, social support was a key theme including family, friends, work, peers, social media, and parenting groups (Young et al., 2020). Parents’ internal skills and abilities were also included within the thematic network of parental resilience. The study of Young et al. (2020) suggests that there are similarities between the experiences of new parents and the description of perinatal resilience developed within this research.

### Future Research

This study is a first step in a broader research protocol to enhance resilience during the first 1,000 days. The concept analysis delivers an important foundation for the development of a perinatal resilience model. In the next phase we will compose a battery of instruments to measure perinatal resilience attributes and integrate this into a pilot study to enhance resilience in (future) parents. These findings can in turn inform healthcare providers about the applicability of resilience-based interventions and the opportunities to guide individuals to positive adaptation. Another interesting track for future research is to determine to what extent a resilient response at one point in life may help facilitate further resilience in later life. A further interesting scope is to investigate the effect of perinatal resilience of the early care-giving environment on the development of the child.

## Conclusion

A concept analysis of perinatal resilience is unique. This study described the concept of perinatal resilience within the context of the first 1,000 days of life for the first time. We described perinatal resilience as a circular process toward a greater well-being in the form of personal growth, family balance, adaptation or acceptance, when faced with stressors, challenges or adversity during the perinatal period. The presence of resiliency attributes such as social support, sense of mastery, self-efficacy and self-esteem enhance the capacity to be resilient and prevent mental health problems.

## Data Availability Statement

The data used and/or analyzed during the current study are available from the corresponding author on reasonable request.

## Ethics Statement

The studies involving human participants were reviewed and approved by the Commission Medical Ethics Hospital Oost-Limburg, Genk, Belgium. The patients/participants provided their written informed consent to participate in this study.

## Author Contributions

SVH performed the literature review for the concept analysis and created the first version of the concept, devised the methodology, and drafted the manuscript. MB performed the Delphi survey. TN was an independent assessor for the literature review. MB, EF, OT, and AB critically reviewed and edited the manuscript. All authors contributed to the article and approved the submitted version.

## Conflict of Interest

The authors declare that the research was conducted in the absence of any commercial or financial relationships that could be construed as a potential conflict of interest.
