# Supplementary material for: Perinatal Resilience for the First 1,000 Days of Life. Concept Analysis and Delphi Survey
Source: Front Psychol. 2020 Nov 3;11:563432. doi: 10.3389/fpsyg.2020.563432 (PMC7670043; doi:10.3389/fpsyg.2020.563432)
Supplement: Supplementary file 1 [file Table_1.DOCX]

Supplementary Material

# Appendix A – Search string

Pubmed: ((((((((("Gynecology"[Mesh] OR "Obstetrics"[Mesh] OR "Gynecology" [tiab] OR "Obstetrics" [tiab])) OR ("Midwifery"[Mesh] OR "Midwife" [tiab] OR "Midwives" [tiab])) OR ("Maternal Health"[Mesh] OR "Maternal Health" [tiab])) OR ("Prenatal Care"[Mesh] OR "Prenatal care" [tiab] OR "Antenatal care" [tiab])) OR ("Perinatal Care"[Mesh] OR "Perinatal Care" [tiab] OR "Perinatology"[Mesh] OR “Perinatology” [tiab])) OR ("Maternal Health Services"[Mesh] OR "Maternal health services" [tiab]))) AND ((((((("Pregnant Women"[Mesh] OR "Pregnant Woman" [tiab] OR "Pregnant women" [tiab] OR "Expectant mother" [tiab])) OR ("Pregnancy"[Mesh] OR "Pregnancy" [tiab] OR "Pregnancy Trimesters"[Mesh] (explode) OR "Pregnancy Trimesters" [tiab] OR "Pregnancy second trimester" [tiab] OR "Pregnancy third trimester" [tiab] OR "Pregnancy first trimester" [tiab])) OR ("Delivery, Obstetric"[Mesh] OR "Obstetric delivery" [tiab] OR "obstetric deliveries" [tiab])) OR ("Parturition"[Mesh] OR "Parturition" [tiab] OR "Childbirth" [tiab] OR "Childbirths" [tiab] OR "Birth" [tiab] OR "Births" [tiab])) OR ("Postpartum Period"[Mesh] OR "Postpartum period" [tiab] OR "Postnatal Period" [tiab] OR "Post-natal period"[tiab] OR "Puerperium" [tiab] OR "Postpartum" [tiab] OR "After birth" [tiab])) OR ("Peripartum Period"[Mesh] OR "Peripartum period" [tiab] OR "perinatal period" [tiab] OR "antenatal period" [tiab] OR "Prenatal period" [tiab]))) AND ("Resilience, Psychological"[Mesh] OR "Resilience" [tiab])

Embase: ('psychological resilience'/exp OR 'psychological resilience' OR 'resilience'/exp OR 'resilience' OR 'resiliency'/exp OR 'resiliency') AND ('gynecology'/exp OR 'gynecology' OR 'obstetric delivery'/exp OR 'obstetric delivery' OR 'midwife'/exp OR 'midwife' OR 'maternal care'/exp OR 'maternal care' OR 'prenatal care'/exp OR 'prenatal care' OR 'perinatal care'/exp OR 'perinatal care' OR 'perinatology'/exp OR 'perinatology' OR 'maternal health service'/exp OR 'maternal health service' OR 'postnatal care'/exp OR 'postnatal care' OR 'midwifery'/exp OR 'midwifery' OR 'obstetrics'/exp OR 'obstetrics' OR 'midwives'/exp OR 'midwives' OR 'maternal health'/exp OR 'maternal health' OR 'antenatal care'/exp OR 'antenatal care') AND ('first trimester pregnancy'/exp OR 'first trimester pregnancy' OR 'second trimester pregnancy'/exp OR 'second trimester pregnancy' OR 'third trimester pregnancy'/exp OR 'third trimester pregnancy' OR 'pregnant woman'/exp OR 'pregnant woman' OR 'pregnant women'/exp OR 'pregnant women' OR 'pregnancy'/exp OR 'pregnancy' OR 'expectant mother'/exp OR 'expectant mother' OR 'pregnancy trimesters'/exp OR 'pregnancy trimesters' OR 'pregnancy second trimester'/exp OR 'pregnancy second trimester' OR 'pregnancy third trimester'/exp OR 'pregnancy third trimester' OR 'pregnancy first trimester'/exp OR 'pregnancy first trimester' OR 'obstetric delivery'/exp OR 'obstetric delivery' OR 'obstetric deliveries' OR 'parturition'/exp OR 'parturition' OR 'childbirth'/exp OR 'childbirth' OR 'childbirths' OR 'birth'/exp OR 'birth' OR 'births' OR 'perinatal period'/exp OR 'perinatal period' OR 'postpartum period'/exp OR 'postpartum period' OR 'postnatal period'/exp OR 'postnatal period' OR 'post-natal period' OR 'puerperium'/exp OR 'puerperium' OR 'postpartum'/exp OR 'postpartum' OR 'after birth' OR 'peripartum period'/exp OR 'peripartum period' OR 'antenatal period'/exp OR 'antenatal period' OR 'prenatal period'/exp OR 'prenatal period')

Web of Science: (“Gynecology" OR "Obstetrics" OR "Midwifery" OR "Midwife" [tiab] OR "Midwives" OR "Maternal Health" OR "Prenatal Care" OR "Antenatal care" OR "Perinatal Care" OR "Perinatology" OR "Maternal Health Services" OR “Postnatal Care”) AND ("Pregnant Women" OR "Pregnant Woman" OR "Expectant mother" OR "Pregnancy" OR "Pregnancy Trimesters"[Mesh] OR "Pregnancy second trimester" OR "Pregnancy third trimester" OR "Pregnancy first trimester" OR "Obstetric delivery" OR "obstetric deliveries" OR "Parturition" OR "Childbirth" OR "Childbirths" OR "Birth" OR "Births" OR "Postpartum Period" OR "Postpartum period" OR "Postnatal Period" OR "Post-natal period" OR "Puerperium" OR "Postpartum" OR "After birth" OR "Peripartum Period" OR "Peripartum period" OR "perinatal period" OR "antenatal period" OR "Prenatal period") AND ("Resilience, Psychological" OR "Resilience" OR “Resilient” OR “resiliency”)
